# In vitro comparative quality evaluation of different brands of metformin hydrochloride tablets available in Mekelle City, Tigray Regional State, Ethiopia

**DOI:** 10.1186/s40360-026-01123-1

**Published:** 2026-03-12

**Authors:** Brhane Gebrehiwot Welegebrial, Zenawi Gezae, Samrawit Kidanemariam Gebrekidan, Gebru Gebremeskel Gebrerufael, Haylay Araya Gebregzabiher, Abrahaley Mulu Kidane, Gebretekle Gebremichael Hailesilase

**Affiliations:** 1 Faculty of Pharmacy, College of Health Sciences, Mekelle University, Mekelle, Ethiopia; 2 Department of Pharmacy, College of Medicine and Health Sciences, Adigrat University, Adigrat, Ethiopia; 3 Department of Statistics, College of Natural and Computational Sciences, Adigrat University, Adigrat, Ethiopia

**Keywords:** Metformin hydrochloride, Hardness test, Dissolution profile, Mekelle, Ethiopia

## Abstract

**Background:**

Administration of poor-quality medications can result in various effects that jeopardize the drugs’ safety and effectiveness. Due to a lack of adequate post-market surveillance studies, making decisions regarding the quality of medicines, including metformin hydrochloride tablets, available in Mekelle City is challenging. Thus, the aim of the study was to evaluate the quality of different brands of metformin hydrochloride tablets sold in Mekelle City, Tigray Regional State, Ethiopia.

**Methods:**

A total of eight brands of 500 mg metformin hydrochloride tablets were collected from drug retail outlets in Mekelle City. In vitro quality control tests were conducted based on the WHO visual inspection tool and United States Pharmacopeia (USP) specifications. These included visual inspection, weight variation, thickness, hardness, friability, disintegration time, dissolution, and an assay of active ingredient. One-way ANOVA was used to compare all parameters of quality control. A difference was deemed statistically significant when *P* < 0.05. One-way ANOVA, model-independent approaches, and model-dependent approaches were used to compare the dissolution profiles and to determine the nature of active pharmaceutical ingredient release.

**Results:**

This study showed that all metformin hydrochloride brands marketed in Mekelle City complied with the WHO visual inspection and USP specifications for visual inspection, weight variation, friability, disintegration time, and assay of the active ingredient. Of the samples tested, brand E and brand F exhibited the highest and lowest hardness values, respectively, at 313.88 N and 104.06 N. Moreover, all eight brands evaluated released more than 80% of their drug contents within 30 min as outlined in the USP. However, the difference factor (f_1_) and similarity factor (f_2_) have indicated that only brands A, E, and H were similar (can be used interchangeably) with the comparator product. Weibull release model suits the drug release data, which had the highest correlation coefficient.

**Conclusions:**

All brands met pharmacopoeial specifications, but significant variations in hardness and dissolution profiles were observed, with only three brands showing profile similarity to the comparator. Hence, the finding serves as an alert to examine the dossier evaluations and legal approval processes of the Ethiopian Food and Drug Authority.

## Introduction

Diabetes mellitus is a chronic disease condition that arises when the pancreas fails to generate sufficient insulin or when the body cannot utilize the insulin it produces effectively [1]. It is a major public health concern and ranks among the four primary non-communicable diseases that global leaders are focusing on [2]. From 1990 to 2022, the number of individuals living with diabetes increased from 200 million to 830 million. The increase in prevalence has been more pronounced in low- and middle-income countries compared to high-income countries [1]. Globally, type 2 diabetes made up 90% of the total diabetes prevalence in 2021 [3]. In the previous study, the prevalence of type 2 diabetes mellitus in Ethiopia was 6.5% [4].

**Fig. 1:**
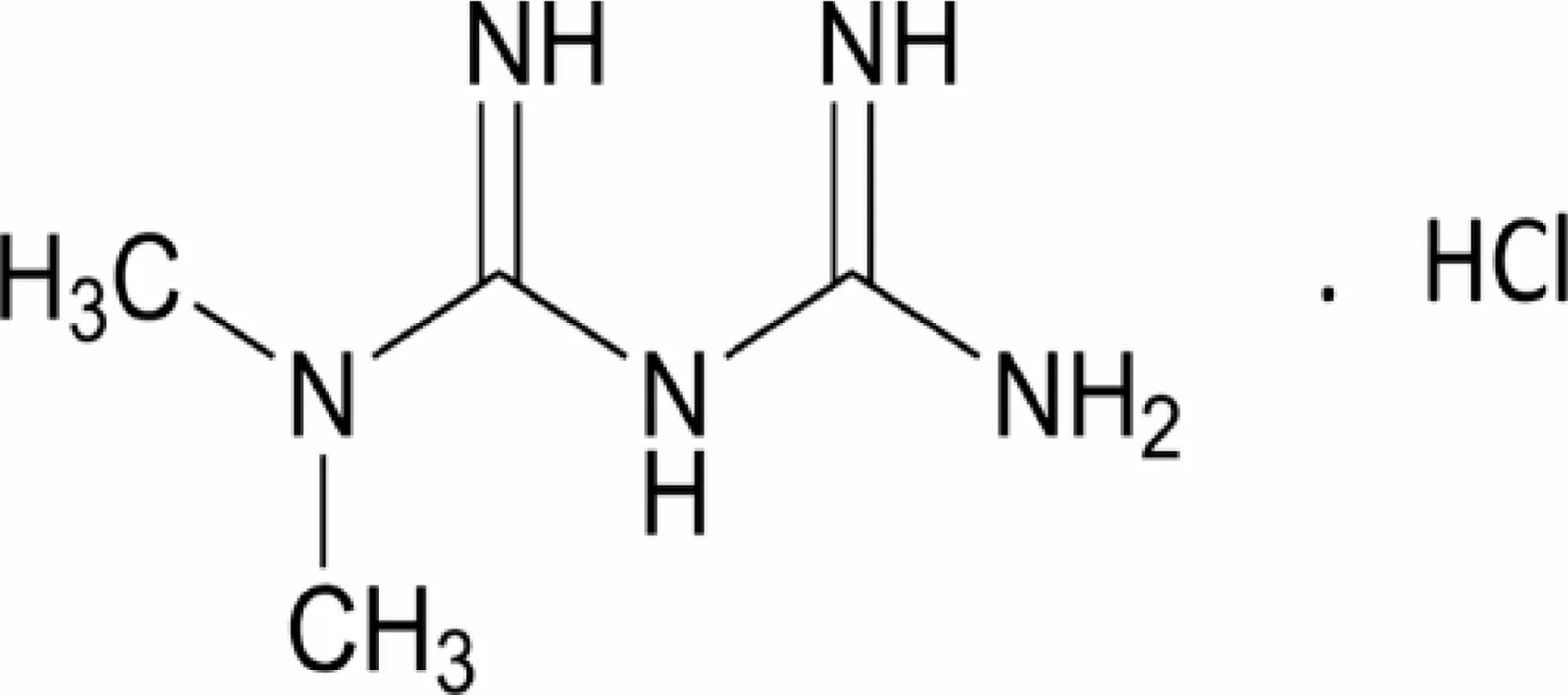
Chemical structure of metformin hydrochloride

Metformin hydrochloride is the most frequently used medication for managing type 2 diabetes mellitus [5, 6]. It is among the essential medications listed in the World Health Organization (WHO) essential medicine list [7]. Metformin hydrochloride is a biguanide-class oral antidiabetic agent [8]. It was chemically identified as N, N-dimethyl-imido-dicarbonimidic diamide hydrochloride (Fig. 1). It is poorly soluble in lipid media. This results in the drug having a low capacity to penetrate the cell membrane [9]. At physiological pH (with a pKa of about 11.5), metformin primarily exists as a cation [10].

Metformin hydrochloride, which is classified as class III by the Biopharmaceutical Classification System, is a drug that has high permeability but low solubility [11, 12]. Hence, the permeability is the step that limits the rate of drug absorption. The vital quality control factor for drugs is dissolution, since it affects absorption directly. The WHO acknowledges the potential to waive in vivo bioequivalence studies for immediate-release, solid oral dosage forms containing active pharmaceutical ingredients (APIs) classified as classes I and III according to the Biopharmaceutical Classification System, based on comparative in vitro dissolution studies [13].

Multisource products, in the form of generic drug products, were globally introduced to provide alternatives to specific brands in regions where they are limited or too costly for the populations especially in low-income countries. Generic medicines have the same active ingredient, strength, route of administration, safety profile, dosage form, efficacy, quality standards, intended use, and are bioequivalent to brand-name medicines. Therefore, they are generally meant to be interchangeable with the branded medicine [14]. However, this trend has also accompanied to a rise in the prevalence of fake, substandard, and counterfeit drug products [15]. According to the WHO 2017 report, as much as 10% of medications in low- and middle-income countries could be substandard or falsified, resulting in a cost of $30 billion each year to the global health system [16]. Additionally, Asrade et al. carried out a systemic review in which they found that 22.6% of the medicines available in Africa are either falsified or substandard [17]. Even though comprehensive data on the quality of medicines in Ethiopia is lacking, there are studies indicating the presence of poor-quality medicines in the country. A study conducted by Hassen et al., for example, found that 21.4% of the 679 samples tested did not fulfil the required quality standards [18].

Besides, studies carried out in different parts of Ethiopia on various pharmaceutical products demonstrated the existence of poor-quality drugs in the market [19–27]. Furthermore, different studies done specifically on quality of different brands of metformin hydrochloride tablets in Ethiopia show there are products that do not fulfill the required quality specifications or products that were not bioequivalent with the comparator product. For instance, a study conducted in Addis Ababa by Kasahun et al. found that one out of five analyzed brands of metformin hydrochloride tablets did not meet the required quality standards [27]. Another study done by Tesfay et al. to evaluate quality of various brands of metformin hydrochloride sold in western and northwestern Tigray, Ethiopia, found that four brands were not bioequivalent to the comparator [28]. The results indicated that poor-quality medicines could put the Ethiopian health system at risk.

Thus, it is necessary to continuously evaluate the pharmaceutical quality of essential drugs available in the market to guarantee that these medicines are of high quality.

Despite the fact that it is the duty of the Ethiopia Food and Drug Authority (EFDA) to regulate the quality of pharmaceuticals in circulation within the nation, there may be a low-quality drug products in the country, likely due to resource limitations and the extent of post-marketing surveillance activities. This may result in the manifestation of illegal acts in the country, taking advantage of weaknesses in the legal structure (24). Moreover, weak regulatory enforcement, inadequate control of the informal market, ineffective port management, poor inter-agency cooperation, and limited resources were some of the reasons for proliferation of poor-quality drug products in Ethiopia [29]. When patients take poor-quality medication, it can result in various effects that jeopardize the drugs’ safety and effectiveness. Due to a lack of adequate post-market surveillance studies, making decisions regarding the quality of medicines, including metformin hydrochloride tablets, available in Mekelle City is challenging. Considering the issue mentioned above, the aim of the study was to evaluate the quality of different brands of metformin hydrochloride tablets sold in Mekelle City, Tigray Regional State, Ethiopia.

## Methods and materials

### Study area and period

The samples were collected from drug retail outlets located in Mekelle City, within the Tigray Regional State of Ethiopia from January January 01, 2025 to January 31, 2025. Mekelle is found in northern Ethiopia, around 783 km from the capital city, Addis Ababa. The city serves as a commercial center for pharmaceutical transactions in the Tigray region. It has an estimated population of about 528,897 residents; the city is divided into seven sub-cities [30]. The Mekelle City health office report indicates that the city has one governmental comprehensive specialized hospital, two general hospitals, thirteen primary hospitals, eleven health centers, one hundred thirty-nine clinics, and three hundred-seven drug retail outlets (one hundred fifty-nine pharmacies, and one hundred forty-eight drug stores). The study’s experiment took place in the quality control laboratory of Addis Pharmaceutical Factory (APF) from May 1, 2025 to May 31, 2025. APF is a pharmaceutical manufacturing factory located in the city Adigrat Town, within Ethiopia’s Tigray regional state, equipped with various analytical instruments such as ultraviolet (UV)-visible spectrophotometers, dissolution testers, and disintegration testers for sample analysis.

### Study design

An in vitro experimental study design was employed to assess the quality of different brands of metformin hydrochloride tablets marketed in Mekelle City.

### Instruments and equipment

Analytical balance (CAP124S, Sartorious AG, Germany); disintegration apparatus (Pharma-test, Germany); hardness tester (DW-THT-1350, Drawell, China); thickness tester (CD-8, ASX, Japan); PH meter (Metler Toledo, Switzerland); dissolution apparatus (Pharma-test, Germany), UV-visible spectrophotometer (Cary 60 UV-VIS, Agilent Technologies, United States); friability tester (FAP-2A, United States); and 0.45 μm Nylon membrane filter were used.

### Chemicals and reagents

Distilled water; sodium hydroxide (analytical grade, Sigma-Aldrich, India); and potassium dihydrogen orthophosphate (analytical grade, Sisco Research Laboratories Pvt. Ltd., India) were used for the study. All chemicals and reagents used in this study were analytical grade. A secondary reference material of metformin hydrochloride was kindly obtained from APF (Adigrat, Ethiopia).

### Sampling techniques and sample collection

Eight brands of metformin hydrochloride tablets, each claiming to contain 500 mg, were collected from licensed drug retail outlets in Mekelle City. The drug retail outlets were selected using a convenience sampling technique, while the mystery shopper approach was contemplated for sample collection. Even with the existence of a functioning national medicines regulatory system in Ethiopia, gaps in regulatory coverage and enforcement during distribution may allow some drug outlets to stock unregistered pharmaceutical products. This situation could lead to feelings of suspicion and anxiety among outlet employees regarding investigations. Hence, mystery shopping method were employed. Visits were made to nearly all drug retail outlets in Mekelle City, resulting in the acquisition of only eight brands of metformin hydrochloride tablets with a strength of 500 mg. When a drug outlet had multiple brands, all of those different brands were collected. Once all samples had been gathered, they were kept under the storage conditions specified on the product label until experimental analysis was performed. Finally, the product’s quality was evaluated as per the guidelines of the WHO visual inspection tool and United States Pharmacopeia (USP). As Glucophage, the metformin innovator product, was unavailable in drug retail outlets during the study period, one of the tested brands (brand B) was chosen as the comparator product. The product used for comparison was chosen in accordance with the WHO Guideline. This guidance indicates that the selected product should be one that has received approval in an ICH-associated country and is the market’s leading brand [31]. Thus, brand B was selected as the comparator product, having fulfilled this criterion. Table 1 provides detailed information about the metformin hydrochloride tablets that are included in this study.

### Methods

For each of the eight brands of 500 mg metformin hydrochloride tablets collected from Mekelle City, in vitro quality control attributes were assessed. These included visual inspection, weight variation, thickness, hardness, friability, disintegration time, dissolution, and assay of active ingredient. The methods outlined in the USP and WHO visual inspection tool were followed to conduct the study [32, 33].

#### Visual inspection

Initially, the registration status of each collected sample was verified through the registration database, part of the EFDA’s Electronic Regulatory Information System (ERIS). Then the samples were visually inspected to assess their physical characteristics (such as shapes, color, breaks, markings, cracks, and splits), packaging, and labeling details (including the API name, country of origin, manufacturing company, storage instructions, production date, expiry date, lot number, number of units per strip/packages, and medicine strength) according to WHO standards [33].

**Table 1:** A detailed label information and price per tablet of different brands of metformin hydrochloride 500 mg tablets evaluated in the study area

Code	Brand	Country of origin	Manufacturer	Type of tablet	Batch No.	Mfg. date	Expiry date	Price per tablet in USD	Colour
A	Metformin Denk 500	Germany	DENK PHARMA GmbH &co. KG	Film-coated	29043U2	01/2024	01/2027	0.1185	White
B*	BROT 500 mg	Cyprus	Medochemie Ltd	Film-coated	ABC115	03/2024	03/2029	0.0277	White
C	Nefort	India	Saga Lifescience Limited	Film-coated	NAJOO12401	12/2024	11/2027	0.0237	White
D	MF-500	India	Pil Psychotropics India Ltd.	Uncoated	CLI44006	07/2024	06/2026	0.0237	White
E	Metformin tablets BP 500 mg	India	Medopharm Pvt. Ltd.	Film-coated	222,063,047	07/2022	06/2025	0.0237	White
F	Formentin 500	Ethiopia	Glocare Pharma Manufacturing PLC.	Film-coated	TD014	01/2025	12/2027	0.0198	White
G	Gluformin	Ethiopia	Addis Pharmaceutical Factory S.C.	Film-coated	33887	01/2025	01/2028	0.0198	White
H	ME-500	Ethiopia	Humanwell Pharmaceutical Ethiopia PLC.	Film-coated	46240424	27/04/2024	26/04/2026	0.0237	White

Note: B*= comparator product, USD=United States Dollar, 1 USD = 126.53 Ethiopian Birr

#### Weight variation

To make sure that the drug content in each unit dose is distributed within a narrow range around the label strength, a weight variation test is necessary [31]. From each brand of metformin hydrochloride, twenty tablets were selected randomly, weighed separately on an analytical balance, and their average weight was calculated. Then following formula Eq. (1) was used to determine the percentage deviation from the average:

1 $$\begin{aligned}&Percent\:of\:weight\:variation\cr&=\frac{\left(Individual\:weight-Average\:weight\right)}{Average\:weight}\times100\end{aligned}$$

#### Friability test

First, twenty randomly selected tablets from each brand were weighed using analytical balance. Then, the tablets were put inside the friability tester’s drum and rotated at 25 revolution per minute for four minutes. After that, the tablets were reweighed following a hundred revolutions and de-dusting. Lastly, the following formula Eq. (2) was used to determine the percentage of friability:

2 $$\begin{aligned}{\%}\:\mathrm{o}\mathrm{f}\:\mathrm{f}\mathrm{r}\mathrm{i}\mathrm{a}\mathrm{b}\mathrm{i}\mathrm{l}\mathrm{i}\mathrm{t}\mathrm{y}&=\frac{\left(\mathrm{I}\mathrm{n}\mathrm{t}\mathrm{i}\mathrm{t}\mathrm{i}\mathrm{a}\mathrm{l}\:\mathrm{w}\mathrm{e}\mathrm{i}\mathrm{g}\mathrm{h}\mathrm{t}-\mathrm{F}\mathrm{i}\mathrm{n}\mathrm{a}\mathrm{l}\:\mathrm{w}\mathrm{e}\mathrm{i}\mathrm{g}\mathrm{h}\mathrm{t}\right)}{\mathrm{I}\mathrm{n}\mathrm{i}\mathrm{t}\mathrm{i}\mathrm{a}\mathrm{l}\:\mathrm{w}\mathrm{e}\mathrm{i}\mathrm{g}\mathrm{h}\mathrm{t}}\cr&\quad\times100\end{aligned}$$

#### Hardness test

Ten tablets from each brand were selected for this test, and a hardness tester was used to determine how hard each tablet was. The crushing strength that breaks a tablet was measured after each tablet was positioned between two anvils and force was applied to the anvils.

#### Thickness test

Ten tablets from each brand were tested for thickness, and the mean and standard deviation were then calculated.

#### Disintegration time test

Disintegration time test was carried to determine the time required for the tablets to disintegrate. Using a disintegration tester, the time needed to disintegrate of six randomly chosen tablets per brand was measured at a temperature of 37 ± 2 °C in 900 mL of distilled water. The time it took for the tablet to break apart and go through the mesh was noted. The average time for disintegration was then calculated.

#### Assay

Drug content determination helps to assure the presence of the required amount of active ingredient as claimed by the manufacturer [12]. The USP states that metformin hydrochloride tablets should contain not less than 95.0% and not more than 105.0% of the stated amount. Assay test was done using UV-visible spectrophotometer.

##### Sample solution preparation

Firstly, twenty tablets of metformin hydrochloride from each brand were weighed and powdered using a mortar and pestle. Then a quantity of the powder equivalent to 0.1 g of metformin hydrochloride was shaken with 70 mL of distilled water for 15 min, and made the volume to 100 mL with distilled water. After filtering the solution, 10 mL of the filtrate was diluted to 100 mL with distilled water. Then 10 mL of the resulting solution was further diluted to 100 mL with distilled water to give a nominal concentration of 10 µg/mL.

##### Standard solution preparation

A solution of metformin hydrochloride reference standard having a known concentration of 10 µg/mL in water was prepared using 0.1 g of the reference standard of in similar manner with the preparation of sample.

Then, absorbances of both the resulting standard and sample solutions were determined concomitantly, in 1 cm, at a wavelength of 232 nm by UV-visible spectrophotometer, using water as blank. The percentage of the labeled amount of metformin hydrochloride in each brand was calculated using the following equation (Eq. 3):

3 $$Assay\left(\%\right)=\left(\frac{{A}_{u}}{{A}_{s}}\right)\times\left(\frac{{C}_{s}}{{C}_{u}}\right)\times100$$

Where, A_u_: is Absorbance of the sample solution

As: is absorbance of the standard solution

Cs: is concentration of metformin hydrochloride in the standard solution

Cu: is concentration of metformin hydrochloride in the sample solution

#### Dissolution test

First calibration curve was developed using the following procedures: a stock solution was prepared by dissolving 100 mg of metformin hydrochloride reference standard in 100 ml of phosphate buffer (at pH 6.8). From the stock solution, six concentration levels (1 µg/ml, 2 µg/ml, 3 µg/ml, 4 µg/ml, 5 µg/ml, 6 µg/ml) were prepared with phosphate buffer. Absorbances of these concentrations were determined using UV- visible spectrophotometry at wavelength of 233 nm. Then, concentrations of metformin hydrochloride (x-axis) against absorbance (y-axis) was plotted to obtain the calibration curve (y = mx+b).

Dissolution test of metformin hydrochloride tablets was done using dissolution apparatus type II (paddle apparatus) in a dissolution medium of phosphate buffer (PH = 6.8). The water bath was filled up to the required level and six dissolution vessels were filled with 1000 ml dissolution medium. The temperature of the medium was maintained at 37 ± 0.5 °C. Six tablets were randomly selected from each brand and placed in separate dissolution vessels. The dissolution was carried out for 45 min. At the specified intervals (5, 15, 30 and 45 min), filtered 10 ml samples were withdrawn and replaced with an equal amount of blank dissolution medium maintained at same temperature.

The withdrawn samples were diluted to 100 fold (1 ml to 100 ml) and their absorbances were measured at 233 nm wavelength using UV- visible spectrophotometer using phosphate buffer (PH = 6.8) as blank. Lastly, the percent of drug release each sample at (5, 15, 30, 45 min) was determined from the calibration curve. The release of API from a dosage form in a dissolution medium was presented graphically, which was a plot of percentage of drug release against time. Under the chosen condition sets, it displays the API release pattern.

In the current study, one-way analysis of variance (ANOVA), model-independent approaches (similarity factor (f_2_), difference factor (f_1_), dissolution efficiency (DE), and mean dissolution time), as well as model-dependent approaches (zero-order, first-order, second-order, third-order, Hixson–Crowell, Higuchi, Korsmeyer-Pepas, and Weibull model) were used to compare the dissolution profiles and to determine the nature of API release (Table 2).

**Table 2:** Model dependent and model independent parameters for comparison of dissolution profiles of different brands of metformin hydrochloride tablets

Model-dependent	Model-independent
Zero-order ${Q}_{t}={K}_{0}.t+{Q}_{0}$	${f}_{1}=\left\{\left[\frac{\sum_{t=1}^{n}\left|{R}_{t}-{T}_{t}\right|}{\sum_{t=1}^{n}{R}_{t}}\right]\right\}100$
First-order $$\mathrm{log}\:{Q}_{t}=\mathrm{log}\:{Q}_{0}+\frac{{K}_{1}t}{2.303}$$	$${f}_{2}=50\:\mathrm{log}\left\{\left[\frac{100}{\sqrt{\left[1+\sum_{t=1}^{n}\left\{{R}_{t}-{T}_{t}\right\}\raisebox{1ex}{$2$}\!\left/\!\raisebox{-1ex}{$n$}\right.\right]}}\right]\right\}$$
Second-order $\frac{1}{Q}=K.t+\frac{1}{{Q}_{0}}$	$DE=\frac{{\int}_{{t}_{1}}^{{t}_{2}}ydt}{y100({t}_{2}-{t}_{1})}\times100$
Third-order $\frac{1}{Q}=K.t+\frac{1}{{Q}_{0}^{2}}$	$MDT=\frac{{\sum}_{j=1}^{n}\:{t}_{j}^{AV}X\varDelta{Q}_{j}}{\sum_{j=1}^{n}\varDelta{Q}_{j}}$
Hixson–Crowell model ${Q}^{\frac{1}{3}}=K.t-{Q}_{0}^{\frac{1}{3}}$	
Higuchi model $Q=K.\sqrt{t}$	
Korsmeyer-Peppas $$\raisebox{1ex}{${M}_{t}$}\!\left/\!\raisebox{-1ex}{${M}_{\infty}$}\right.=ktn$$	
Weibull model $m=1-\mathrm{e}\mathrm{x}\mathrm{p}\left[\frac{-{\left(t\right)}^{b}}{a}\right]$	
Best model criteria ${r}^{2}=\frac{{\left[N.{\sum}_{i=1}^{N}{X}_{i}.{Y}_{i})-\sum_{i=1}^{N}{X}_{1}\sum_{i=1}^{N}{Y}_{i}\right]}^{2}}{\left[N.\sum_{i=1}^{N}{X}_{i}^{2}-{\left(\sum_{i=1}^{N}{X}_{i}\right)}^{2}\right].\left[N.\sum_{i=1}^{N}{Y}_{i}^{2}-{\left(\sum_{i=1}^{N}{Y}_{i}\right)}^{2}\right]}$	
Where, Q_0_ is the initial amount of drug substance Q is the amount of drug substance released at time, t t is time m is the amount of drug substance dissolved at time, t a is time constant b is shape parameter r^2^ is correlation coefficient Y_i_ is observed value i is data point N is number of data points K, K_o_ and K_1_ are rate constants N is number of data points and Mt/M*∞* is the amount of drug released at time t/the amount of released at infinity time t	Where, f_1_ is difference factor f_2_ is similarity factor n is the number of time points R_t_ is the dissolution value of the reference at time, t T_t_ is the dissolution value of the test drug at time, t MDT is the mean dissolution time DE is dissolution efficiency MDT is mean dissolution time ΔQ = Q (t)–Q (t-1), tjAV=(ti + ti-1)/2, and n is amount of time points

#### Data analysis and presentation

The data were analyzed using Microsoft Excel 2016 and Statistical Package for Social Sciences (SPSS version 25). The release of drug substances from the tablets was determined using the Kinet DS version 3.0 software program. One-way ANOVA at 95% confidence interval (CI) was used to compare the average weight, thickness, hardness, friability, disintegration and assay of active ingredient of different brands. Differences were deemed statistically significant when *P* < 0.05. To compare the in vitro dissolution profiles of the various brands of metformin hydrochloride, one-way ANOVA, as well as model-independent and model-dependent methods, were employed. The results were summarized and presented through text, tables and figures.

## Results and discussion

The purpose of this study was to evaluate the quality of metformin hydrochloride tablets available in Mekelle City. All accessible brands of metformin hydrochloride tablets underwent quality control testing to ascertain their quality. Out of the eight different brands of metformin hydrochloride 500 mg tablets collected, three of the brands were manufactured by local companies, and the remaining five brands were imported from foreign countries. At the time of evaluation, none of the products had reached the end of their shelf life. Visual inspection and the tablets’ in vitro quality control tests (weight variation, hardness, friability, thickness, disintegration time, dissolution, and assay of active drug content) were used to evaluate the quality of each sample that was collected.

Although the drug preparation contains the right amount of the active ingredient, this information is insufficient to determine whether the drug is authentic [34]. Visual inspection involves examining the drug’s physical appearance within its packaging and serves as a useful preliminary indicator of product quality [35]. As a result, visual inspection of the product and its packaging is necessary. Visual inspection of the tablets yielded qualitative information on their looks or product labeling (looking for any kind of visual quality problems). Among the eight brands analyzed in this study, brand D was an uncoated tablet, while the other seven brands were film-coated tablets.

All of the brands were white in color and came in blister packaging with all the information that was required printed on them (Table 1). Additionally, the blisters provided a thorough description of the product’s strengths. None of the evaluated brands had any dirt, foreign matter, scratches, cracks, deformations, odd colors, discolorations, or other defects. When the physical characteristics, packaging, and labeling of the products were examined, no signs of fake, mislabeled, or counterfeit products were found (Table 3). Therefore, the visual inspection of all products meets the specifications required by the WHO [33]. In addition, it was found that all of the collected samples were registered in the country ERIS database of the pharmaceutical regulatory body (EFDA).

The dosage uniformity test for tablet dosage forms can be conducted using either the weight variation test or the content uniformity test, and there are specific criteria for choosing one method over the other. USP advises that for uncoated and film-coated tablets with a drug substance weight and ratio of ≥ 25 mg and ≥ 25%, a weight variation test should be conducted; otherwise, a content uniformity test should be performed [32]. Hence, weight variation test were carried out for this particular study. In addition to showing the consistency of the product’s API content, the weight variation test is an indicator of the manufacturers’ compliance with good manufacturing practices. The amount of API in the finished pharmaceutical products can be directly affected by weight variation in the tablet formulation, which can occur for a number of reasons and lead to poor content uniformity in the dosage forms. There should be as minimum weight variation between the tablets as possible to guarantee a consistent dosage. This could lessen the possibility of getting an underdose or overdose, which could have unpredictable therapeutic effects [36].

According to the USP specification, which states that if the strength of the tablet is greater than 324 mg and no more than two of the individual tablet weights deviate from the average weight by more than ± 5% and none deviate by more than ± 10%, the tablet passes the weight variation test [32]. Thus, in this study, all brands had acceptable weight variation. Two tablets from brand E had a weight variation of more than ± 5% in the current study. However, there was no single tablet of any brand that varied by ± 10% (Table 4). Hence, it fulfills the USP allowed limit for weight variation. Similar results were reported from studies conducted in Saudi Arabia [36] and Ethiopia [26].

**Table 3:** Visual inspection of the packaging and labeling information of tested metformin hydrochloride tablets brands

Sample code	Trade/Brand Name	Manufacturer’s Name and Logo	Manufacturer’s Full Address	Medicine Strength (mg/unit)	Dosage Form	No. of Units per Container	Dosage Statement	Batch/Lot No.	Manufactory and Expiry Date	Storage information	Leaflet or package insert
A	Yes	Yes	Yes	Yes	Yes	Yes	Yes	Yes	Yes	Yes	Yes
B	Yes	Yes	Yes	Yes	Yes	Yes	Yes	Yes	Yes	Yes	Yes
C	Yes	Yes	Yes	Yes	Yes	Yes	Yes	Yes	Yes	Yes	Yes
D	Yes	Yes	Yes	Yes	Yes	Yes	Yes	Yes	Yes	Yes	Yes
E	Yes	Yes	Yes	Yes	Yes	Yes	Yes	Yes	Yes	Yes	Yes
F	Yes	Yes	Yes	Yes	Yes	Yes	Yes	Yes	Yes	Yes	Yes
G	Yes	Yes	Yes	Yes	Yes	Yes	Yes	Yes	Yes	Yes	Yes
H	Yes	Yes	Yes	Yes	Yes	Yes	Yes	Yes	Yes	Yes	Yes

A one-way ANOVA with a 95% CI was also used to statistically compare the weight of tablets. There were significant mean weight differences among the brands (*P* < 0.05). The formulation condition, including mixing, the granulation process, which includes mass production equipment, and the amount of excipients used, may have contributed to the inconsistency in tablets weight among the brands [34]. Then patients may have pharmacodynamic and pharmacokinetic fluctuation after consuming tablets with varying weights [37].

**Table 4:** Results of weight variation of metformin hydrochloride tablets marketed in Mekelle City (*N* = 20)

Brand Code	*Average wt. (g) ± % SD	Minimum % wt. variation	Maximum % wt. variation	Number of tablets with ≥ 5% wt. variation	Number of tablets with ≥ 10% wt. variation
A	0.648 ± 0.005	-1.235	1.698	0	0
B	0.672 ± 0.004	-1.080	1.543	0	0
C	0.544 ± 0.008	-3.125	2.574	0	0
D	0.625 ± 0.003	-0.960	0.960	0	0
E	0.594 ± 0.014	-7.576	6.229	2	0
F	0.643 ± 0.012	-4.666	3.888	0	0
G	0.658 ± 0.012	-2.128	4.559	0	0
H	0.777 ± 0.007	-2.059	1.931	0	0

Where, *=P−value <0.05 at 95% confidence interval; SD= standard deviation; wt. = weight

The friability test is used to assess a tablet’s mechanical strength and resistance to physical shocks that it may encounter during packaging, handling and transportation [38]. Besides, to guarantee patient acceptance, the tablets should be manufactured with reasonable friability and adequate hardness. According to the USP, percentage of friability of tablets should not exceed 1% [32]. In this study, all of the evaluated brands complied with this specification and exhibited friability of less than 1%. Of all the analyzed samples, brand D had the highest percentage friability (0.224%), while brand H had the lowest percentage friability (0%) (Table 5). Among the tested brands, brand D exhibited the highest friability, likely due to it being the only uncoated tablet, whereas all other brands were film-coated. This coating could make these tablets resistant to abrasion. This study demonstrated the mechanical and physical stability of metformin hydrochloride products. Similar results were reported from Ethiopia Gondar [26] and Libya [39], which evaluated different brands of metformin hydrochloride tablets and found a percentage friability of all samples less than 1%. Nevertheless, the results of a one-way ANOVA indicated that the average weight loss associated with the different brands of metformin hydrochloride tablets assessed in the study area did differ statistically significantly (*P* < 0.05). The type and quantity of the binder, the manufacturing process, and the amount of compression force used during the manufacturing process all have a direct impact on the strength of the tablet [19]. Since coating gives tablets additional strength and prevents them from being chirped, the friability test is often only advised for uncoated tablets. However, all of the tablets in this study (coated and uncoated) were put through the test, and they all passed the pharmacopoeial requirements.

**Table 5:** Results of friability of metformin hydrochloride tablets marketed in Mekelle City (*N* = 20)

Brand code	W_1_ in g	W_2_ in g	Weight loss	*Friability (%)
A	12.986	12.983	0.003	0.023
B	13.447	13.445	0.002	0.015
C	10.92	10.919	0.001	0.009
D	12.501	12.473	0.028	0.224
E	11.904	11.903	0.001	0.008
F	12.882	12.878	0.004	0.031
G	13.149	13.148	0.001	0.008
H	15.564	15.564	0	0.000

Where, *=P−value <0.05 at 95% confidence interval; W1 = initial weight; W2 = final weight; g = gram

Tablets should be sufficiently hard to avoid damage during packaging, handling, and transportation. A tablet’s bioavailability, friability, and disintegration time are all affected if its hardness is beyond a specific limit; the less hard the tablet, the more friable it is and the faster it disintegrates [20]. In contrary, it will not disintegrate readily and may lead to failure to meet the dissolution specification, if the tablet is very hard [40]. The result of the hardness test in this study showed that all brands passed the test, which needs to be greater than 4 kg (39.2 N) [41]. However, the average hardness of the products is different from each other, i.e. higher (brand E = 313.88 ± 38.42 N) and lower (brand F = 104.06 ± 5.04 N) (Table 6). Moreover, according to one-way ANOVA statistics, the average tablet hardness of the metformin hydrochloride brands examined in the study area differed significantly (*P* < 0.05). This variation in tablet hardness between brands may be caused by a variety of reasons, including differences in binder, lubricants, granulation method, particle size distribution, and machine speed used [42].

Anomalies pertaining to the weight and dosage uniformity of tablets that would have started earlier in the production process can be detected by periodic evaluations of the dimensions, such as thickness, of tablets [35]. Moreover, a consistent tablet thickness is a crucial characteristic that may have a significant impact on patients’ compliance with medications [43]. USP stated that the thickness of tablets in a batch should fall within a ± 5% variation from the required thickness [32]. In the current study, maximum and minimum average thickness was found with brand B (6.187 ± 0.03 mm) and brand D (4.156 ± 0.10 mm), respectively (Table 6). Brand D, the only uncoated tablet from the tested brands, demonstrated the lowest thickness. This is likely due to its status as the sole uncoated option, while all other brands were film-coated. This coating could cause these tablets to become thicker. The research demonstrated that the thickness of the tablets was close to one another, as the maximum standard deviation was 0.10. As a result, the results are in accordance with USP specifications. Nonetheless, the statistical analysis using one-way ANOVA revealed a significant variation in the mean percentage thickness between tablets of different brands (*P* < 0.05). This difference might be from variations in the manufacturing processes used by different manufacturers, such as fill weight and pressure applied during tablet compression [40].

As the rate-determining stage in drug dissolution and absorption processes, the disintegration of an oral tablet is the main way that a physical change takes place following drug administration. It also aids in predicting the process of tablet fractionation into smaller pieces [19]. Findings of this study revealed that all film-coated (brands A, B, C, E, F, G, and H) and uncoated (brand D) brands passed the disintegration test according to USP, which specifies disintegration time within 30 min for film-coated tablets and 15 min for uncoated tablets (Table 6). Among the analyzed tablets, brand A exhibited the longest average disintegration time of 15.83 ± 0.50 min, whereas brand F had the shortest average disintegration time of 5.48 ± 0.19 min. Additionally, from a statistical perspective, a one-way ANOVA using a 95% CI revealed a significant difference (*P* < 0.05) in average disintegration time among the tested brands. This difference may arise from the fact that various manufacturers use different types and quantities of excipients, as well as differing manufacturing processes [27].

The medication can have the necessary therapeutic effects with few adverse effects on the patient if its API is within the acceptable range [44]. Excessive amount of API will result in overdosing of medication, which increases adverse drug reaction and treatment failure while inadequate amounts of API leading to under-dosing of medication, which will have poor treatment outcomes [45]. The USP’s official monograph states that the API of metformin hydrochloride tablets must not fall below 95% of the label claim or exceed 105% [32]. Findings of the assay test showed that all brands had values that lie within the specified metformin hydrochloride percentage content as stated in the USP. The results of assays presented in Table 6 showed that the average content of metformin hydrochloride ranged from 95.2% (Brand C) to 99% (brand F). However, a one-way ANOVA conducted on the average drug content revealed that the metformin hydrochloride brands examined in this study exhibited a statistically significant difference (*P* < 0.05) in drug content.

**Table 6:** Results of disintegration time, hardness and thickness of metformin hydrochloride tablets marketed in Mekelle City

Brand code	*Hardness (N), (mean ± % SD) (*N* = 10)	*Thickness (mm), (mean ± SD) (*N* = 10)	*Disintegration time (min), (mean ± % SD) (*N* = 6)	*Assay (%)
A	196.41 ± 11.81	5.89 ± 0.03	15.83 ± 0.50	98.34
B	285.77 ± 21.05	6.19 ± 0.03	8.02 ± 0.72	95.61
C	195.76 ± 42.51	5.60 ± 0.08	5.91 ± 0.52	95.20
D	132.23 ± 18.27	4.16 ± 0.10	7.85 ± 0.57	96.01
E	313.88 ± 38.47	5.40 ± 0.05	9.21 ± 0.49	95.43
F	104.06 ± 5.04	4.79 ± 0.05	5.48 ± 0.19	99.01
G	142.13 ± 14.52	5.34 ± 0.10	12.24 ± 0.42	98.94
H	262.72 ± 15.96	5.66 ± 0.06	14.90 ± 0.64	97.28

Where, *=P−value <0.05 at 95% CI, SD = Standard deviation, N = newton, mm = millimeter

In order to evaluate the consistency and release characteristics of product batches, in vitro dissolution testing has become a crucial instrument [46]. One of the in vitro quality control procedures used to forecast the in vivo performance of oral pharmaceutical solid dosage forms, like tablets and capsules, is the dissolution test [21]. It is used for assessing the characteristics and consistency of drug release from a pharmaceutical product [47].

**Fig. 2:**
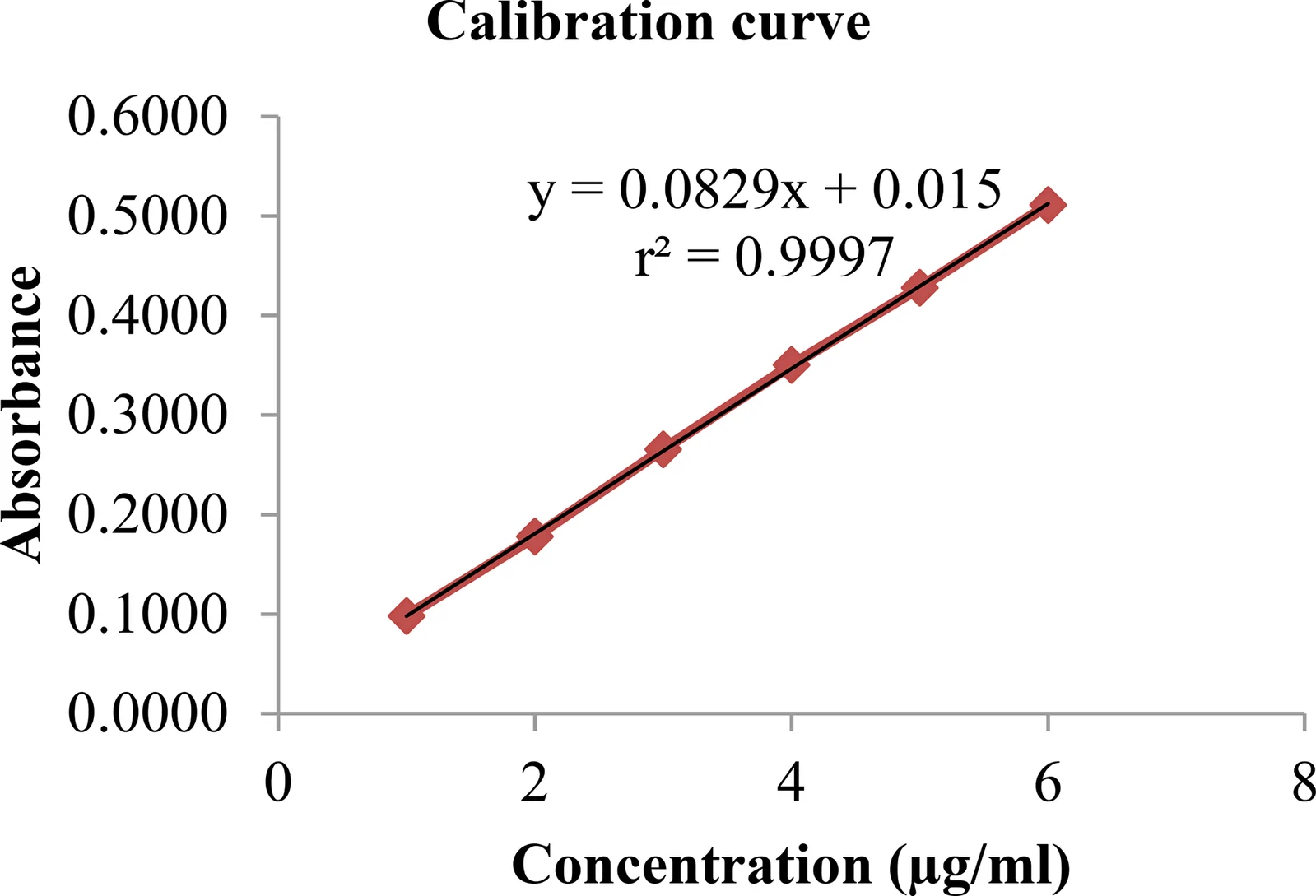
Calibration curve of metformin hydrochloride reference standard in the concentration range of 1–6 µg/ml

The amounts of APIs released from the tablet dosage forms were determined from the calibration curve. A calibration curve, often called a standard curve, is a common method for determining an analyte concentration in an unknown sample by comparing it to a range of known values of the standard solution [19]. In this study a linear equation of y = 0.0829x + 0.015 (where y represents absorbance and x denotes the concentration of the working standard), was constructed to assess the percentage of metformin hydrochloride released. The curve showed a linear correlation between the concentration and the absorbance values with correlation coefficient (r²) of 0.9997 (Fig. 2).

**Fig. 3:**
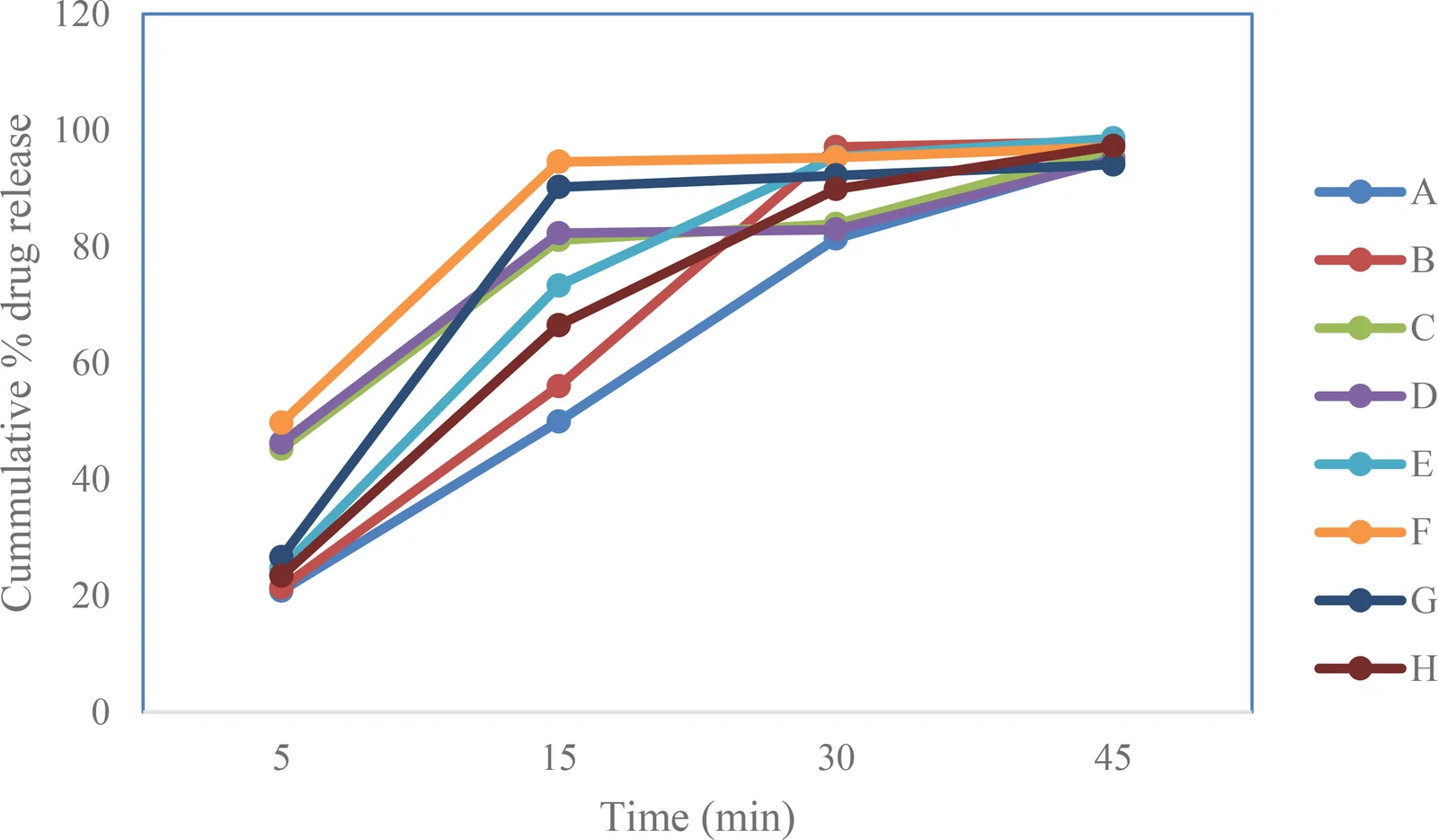
Mean dissolution profiles (% drug release) of eight brands of metformin hydrochloride 500 mg tablets in phosphate buffer pH 6.8. Abbreviations: A=Brand A, B= Brand B, C= Brand C, D=Brand D, E=Brand E, F=Brand F, G=Brand G, H=Brand H

Following the assessment of dissolution testing, different brands of metformin hydrochloride tablets exhibited different drug release patterns at different time intervals, as illustrated in Fig. 3. Among the eight samples, brand A (20.88%) showed the slowest rate of dissolution during the first 5 min. At 5 min, however, brand F (49.73%) were with the highest drug release. Additionally, within 15 min, more than 50% of the drug was released by brands B, C, D, E, F, G, and H. Furthermore, all of the brands released more than 80% of metformin hydrochloride within 30 min, hence, complying with the USP dissolution tolerance limit (Fig. 3; Table 7) [32]. The products released within the range of 81.39% to 97.12% within 30 min. The type and/or quantity of excipients employed, as well as processing and formulation factors, may be the cause of the variation in drug release pattern [21].

**Table 7:** Results of mean percentage drug release of eight brands metformin HCl tablets marketed in Mekelle city

Sampling time (min)	Cumulative % drug release (mean ± SD)							
	A	B	C	D	E	F	G	H
5	20.88 ± 0.04	21.38 ± 0.52	45.25±2.83	46.27±5.33	24.67±1.77	49.73±2.14	26.63±0.78	23.43±1.97
15	49.9±2.09	56.02 ± 4.14	81.12±1.46	82.30±1.92	73.31±1.88	94.57±1.44	90.25±2.16	66.50±2.96
30	81.39 ± 0.61	97.12 ± 2.01	83.88 ± 1.21	82.93± 1.06	95.44±1.55	95.30±0.62	92.24±1.34	89.89±2.39
45	95.30 ± 2.46	98.08 ± 2.39	96.81±1.60	94.88±1.55	98.62±3.58	97.14±1.29	94.12±1.42	97.31±1.79

Comparing dissolution profiles ensures that products performance is comparable and bioequivalent. Most significantly, if the dissolution profile of a comparator product is known, it can enable the required modification in test formulation to attain the same profile of the comparator product and to assess interchangeability of products with the comparator. In this study, to compare the dissolution profiles of different brands of metformin hydrochloride tablets, statistical approaches that included one-way ANOVA, model-independent (f_1_, f_2_, DE, and MDT), and model-dependent parameters were carried out for each brand. Even though all of the tested brands passed the dissolution test, a one-way ANOVA statistical analysis at 95% CI for the pharmacopoeially specified duration of 30 min indicated significant differences in the release patterns of different brands of metformin hydrochloride tablets (*P* < 0.05). The ANOVA was employed solely to assess variations in dissolution among the brands at the pharmacopoeial limit of 30 min. Moreover, to find out if different brands might be used interchangeably with the comparator, the current study employed model-independent parameters (fit factors (f_1_ and f_2)_, DE, and MDT).

Moreover, dissolution profiles of the products were compared by fit factor approaches of f_1_ and f_2_, with the comparator product (brand B). The f_1_ values should be close to 0 (generally values less than 15) and the f_2_ value should be close to 100, with values between 50 and 100 suggest that the two-dissolution profiles are bioequivalent so the products can be interchanged safely [48]. Accordingly, in this study brands C, D, F, and G were not bioequivalent to the comparator (brand B) since their f_1_ value was greater than 15% and the f_2_ was less than 50% (Table 8). With the exception of brands A, E, and H, none of the brands can be considered interchangeable with the comparator product because their f_1_ and f_2_ values are so far from the permitted limit. This is consistent with the study conducted in Western and Northwestern Tigray, Ethiopia, which identified only two brands as bioequivalent to the comparator drug among six analyzed products [28].

Additionally, the current study evaluated the interchangeability of different brands of metformin hydrochloride tablets with the comparator drug using DE. The percentage of DE differences between the comparator and tested medications should be within ± 10% for two drugs that are to be used interchangeably, and vice versa [49]. Results of this study revealed that every brand has a DE difference of less than 3, with the exception of brand F (Table 8). This result aligns with the research done in Jimma, where only one of the ten examined brands was found to be bio-inequivalent with the innovator [12]. This, however, is different from the study conducted in Jordan, where only one brand was found to be interchangeable and three brands were not interchangeable with the comparator product [50]. This difference may arise from the inclusion of different types of brands in the study.

Besides, MDT was determined to ascertain the drug’s rate of dissolution and the time at which the drugs activity began. Determining the amount of drug ingredient released from the dosage form and the retarding ability of the polymer in a pharmaceutical context requires evaluating the MDT [34]. According to this study’s findings, brand F had the lowest MDT (6.78), while brand A had the highest MDT (16.5) compared with the other brands (Table 8). Polymers with a higher capacity to retain drugs are indicated by a higher MDT value, and vice versa [51]. Therefore, brand A had the longest onset of action and delayed drug release, whereas brand F had the fastest onset of action and quickest rate of drug release when compared with the other brands analyzed in the current investigation.

**Table 8:** Model-independent approaches results of different brands of metformin hydrochloride tablets

Brands code	f_2_	f_1_	%DE	Difference of %DE (comparator – test product)	MDT
A	55.64	9.20	60.37	7.48	16.50
B			67.84		13.87
C	38.96	23.29	74.17	-6.33	10.52
D	37.88	25.15	74.03	-6.19	9.89
E	54.92	8.36	72.73	-4.88	11.82
F	33.44	25.55	82.51	-14.67	6.78
G	40.11	17.73	75.94	-8.10	8.69
H	61.59	7.53	68.56	-0.71	13.30

In the current study, to evaluate the model-dependent parameters that it best fits, all in-vitro release test data were fitted to different kinetic equations. After fitting mathematical model-dependent techniques to each individual unit of the dissolution data, the model with the highest r^2^ value was considered the best fit of the release data [48]. As revealed in Table 9, the Weibull release model suits the drug release data quite well, which had the highest r^2^.

**Table 9:** Results of coefficients of model-dependent approaches of different brands of metformin hydrochloride tablets

Model dependent parameters	Metformin hydrochloride tested brands code							
	A	B	C	D	E	F	G	H
Zero-order	0.9531	0.8667	0.7781	0.7355	0.7853	0.5557	0.5582	0.8481
First-order	0.8540	0.7891	0.7155	0.6828	0.6878	0.5436	0.5378	0.7351
Second-order	0.7240	0.6803	0.6586	0.6360	0.6060	0.5338	0.5241	0.6337
Third-order	0.6216	0.5951	0.6123	0.5979	0.5548	0.5263	0.5164	0.5685
Korsmeyer-Pepas	0.9906	0.9647	0.9087	0.8860	0.9136	0.8033	0.7998	0.9407
Weibull model	0.9922	0.9696	0.9361	0.9402	0.9981	0.8529	0.9724	0.9989
Hixson–Crowell model	0.8935	0.8211	0.7361	0.7000	0.7200	0.5473	0.5438	0.7736
Higuchi model	0.9045	0.8521	0.4667	0.2907	0.8702	0.0132	0.6661	0.9047

The price of pharmaceutical products is a significant factor in determining access to reasonably priced medications, especially in low-income countries like Ethiopia [52]. Different brands of metformin hydrochloride 500 mg tablets were sold at different prices in Mekelle City (Table 1). The research found that the brands’ each tablet price varied from 0.0198 United States Dollar (USD) (brands F and G) to 0.1185 USD (brand A). Furthermore, despite the fact that all of the analyzed products satisfied pharmacopoeial criteria, the price of each locally produced metformin hydrochloride tablet was between 0.0198 and 0.0237 USD, whereas imported products were priced at 0.0237 to 0.1185 USD. This suggests that products made locally are cheaper than imported medications, and focus should be directed towards local producers to secure reasonably priced medications, provided their quality is not compromised. From a quality control standpoint, the differences in pricing among the brands did not relate to the products’ quality.

## Limitations of the study

Despite the study’s numerous strengths, it has the following limitations. First of all, the results are not applicable to other medications, dosage forms, or different strengths of metformin hydrochloride tablets. Secondly, the study’s findings may not reflect the entire country because of the limited number of samples collected. Third, impurity profiling was not carried out in this study due to a lack of standards for impurity. Fourth, the assessment of bioequivalence or interchangeability between the comparator and other brands was performed in vitro, although conducting it in vivo would yield results that are more accurate. Fifth, a standardized secondary reference material was utilized for the quantitative analysis in place of a primary reference standard. This was due to the inability to access and afford the cost of the primary standard of metformin hydrochloride, as this study lacks funding from any organization or entity. Therefore, additional studies are necessary to tackle these limitations and achieve a more thorough understanding of the pharmaceutical quality of metformin hydrochloride tablets and other medications available on the market.

## Conclusions and recommendation

The study attempted to assess the in vitro quality control parameters as well as interchangeability of different brands of metformin hydrochloride 500 mg tablets that are sold in Mekelle City. All of the tablets met the necessary quality standards for visual inspection, weight variation, hardness, friability, thickness, disintegration time, dissolution, and assay tests.

However, the dissolution profiles of brands C, D, F, and G were statistically dissimilar to the chosen comparator, which may suggest a potential risk of non-bioequivalence based on f1/f2 factors. Thus, further in vivo studies are advised to verify therapeutic equivalence among the different brands. Moreover, the finding serves as an alert to examine the dossier evaluations and legal approval processes of the EFDA. Therefore, it is necessary to conduct post-market surveillance on the quality of drug products that are available in the market. This is due to the fact that ongoing monitoring of drug quality is crucial for guaranteeing that the pharmaceutical products available in the market are safe, effective, and of good quality.

## Data Availability

All essential data are included in the manuscript.

## Abbreviations

ANOVA: Analysis of variance
APF: Addis Pharmaceutical Factory
API: Active pharmaceutical ingredient
CI: Confidence interval
EFDA: Ethiopia Food and Drug Authority
ERIS: Electronic Regulatory Information System
ETB: Ethiopian Birr
f_1_: Dissimilarity factor
f_2_: Similarity factor
MDT: Mean dissolution time
SD: Standard deviation
r^2^: Correlation coefficient
USD: United States Dollar
USP: United States Pharmacopeia
UV: Ultraviolet
WHO: World Health Organization
