# Correction to: In vitro comparative quality evaluation of different brands of metformin hydrochloride tablets available in Mekelle City, Tigray Regional State, Ethiopia

**DOI:** 10.1186/s40360-026-01202-3

**Published:** 2026-10-13

**Authors:** Brhane Gebrehiwot Welegebrial, Zenawi Gezae, Samrawit Kidanemariam Gebrekidan, Gebru Gebremeskel Gebrerufael, Haylay Araya Gebregzabiher, Abrahaley Mulu Kidane, Gebretekle Gebremichael Hailesilase

**Affiliations:** 1 Faculty of Pharmacy, College of Health Sciences, Mekelle University, Mekelle, Ethiopia; 2 Department of Pharmacy, College of Medicine and Health Sciences, Adigrat University, Adigrat, Ethiopia; 3 Department of Statistics, College of Natural and Computational Sciences, Adigrat University, Adigrat, Ethiopia

**Correction to: BMC Pharmacology and Toxicology (2026) 27:66**

**https://doi.org/10.1186/s40360-026-01123-1**

In this article, the average hardness of brand F was incorrectly published as 10.06 N instead of the correct value 104.06 N, and it has been corrected accordingly in the texts of the Abstract, Results and Discussion sections.

Also, the abbreviation section of the paper, the incorrect abbreviation “EFDA = Ethiopia Food and Drug Administration” is corrected as “EFDA = Ethiopia Food and Drug Authority.”

In the table 1 of the article, the incorrect column title “Price per pack in USD” has been corrected as “Price per tablet in USD.”

